# A Letter to the Editors, from George Gregory, M. D. Physician to the Small-Pox and Vaccination Hospital; Inclosing a Correspondence with Dr. Jenner on the Subject of Small-Pox after Vaccination

**Published:** 1822-09

**Authors:** 


Art. II.—*A Letter to the Editors, from* George Gregory, m.d. Physician to the Small-Pox and Vaccination Hospital; *inclosing a Correspondence with* Dr. Jenner *on the Subject of Small-Pox after Vaccination.*

In the 45th volume of your Journal, (April 1821, p. 277,) a letter was published, addressed by Dr. Jenner to the medical profession generally, relative to vaccination, and more especially in reference to the influence exerted on the progress of the vaccine vesicle by the presence of herpetic and other cutaneous affections. This letter was extensively circulated among the members of the profession last year, and doubtless called forth many replies. Every one must have considered Dr. Jenner as well entitled to whatever information he might possess on the important subject of vaccination; and there is no one among us but must have felt a pleasure in contributing, even in the most trifling degree, to the inquiries of one who has himself so widely extended the boundaries of human knowledge.

It was under this impression that I addressed a letter to Dr. Jenner, in reply to his circular, stating, in a few words, the result of my own observations on the several points to which he had adverted, and on which he appeared desirous to receive information. The opportunity which this correspondence gave me of deriving instruction from Dr. Jenner's extensive acquaintance with the subject of vaccination was, I thought, too good to be lost; and I ventured therefore, towards the close of my letter, to address a few queries to Dr. Jenner on the interesting topics connected with the occurrence of *small-pox after vaccination.* I should never have presumed to intrude these upon the notice of the public, had I not been favoured subsequently with a letter from Dr. Jenner, which cannot, I think, fail to prove very interesting to the profession generally. I have not, indeed, directly received Dr. Jenner's authority for its publication; but I hope he will kindly sanction my making this public use of his communication, when the great interest of the subject is viewed. The points upon which he touches in his letter are every day acquiring a higher degree of importance; and the fruits of Dr. Jenner's experience upon topics of much less consequence will, I may safely venture to say, be at all times gratefully received, and attentively weighed, by his professional brethren.

After offering, in compliance with Dr. Jenner's desire, the result of my own experience on the effect of cutaneous disease upon the progress of the vaccine vesicle, I thus proceeded:

"98, *Great Portland-street; June* 22, 1821.

"I trust, Sir, I shall stand excused if I take the opportunity, which the reply to your letter affords, of requesting some information on a subject which now engrosses, in no ordinary degree, the attention of the public and of the medical profession in the metropolis;—I mean the frequent recurrence of small-pox after vaccination. I have reason to know that many practitioners, who have been accustomed to look up to you with great confidence on all matters connected with vaccination, are anxious to know your opinions on this; and I shall think myself particularly fortunate if I should prove the channel of conveying to them the information of which they are in want. Acceptable as they would be at all times, they are now more than ever desirable, since it has been declared, by the highest professional authority recognized in this country, that the pretensions of vaccination to the merit of a perfect and exclusive security in all cases against small-pox were admitted at first rather too unreservedly; while at the same time it is affirmed, that the *controuling* power of vaccination is next in importance to its preventive influence, and still justifies our high estimation of the value of this great discovery. (*Report of the National Vaccine Establishment, April* 1821.)

"The points, Sir, upon which, in common with many of my professional brethren, I am desirous of having your opinion, are the following:

"1. Whether you have recently had reason to alter, or modify, the sentiments formerly promulgated by yourself respecting the *complete* security afforded by perfect vaccination against the contagion of small-pox?

"2. Whether, in *every* case of small-pox which occurs subsequent to vaccination, you imagine that the vaccine vesicle had been disturbed in its progress by one or other of the circumstances mentioned in your circular, viz.—

A. Preoccupation of the skin, by herpes or some other variety of cutaneous disease.

B. Spurious matter.

C. Robbing the vesicle incautiously of its contents.

D. External violence done to the vesicle during its earlier stages.

"3. Whether any other impediments to perfect vaccination are known to you besides the four now enumerated; and, if so, of what kind are they?

"4. What effect is produced by *revaccination* in individuals in whom the vaccine vesicle was disturbed in its progress by either of the four causes mentioned above?

"5. Whether the failure of a well-conducted revaccination is *decisive* in favour of the first process having been gone through regularly.

"6. Whether in all, or in any considerable proportion of the cases of *imperfect* vaccination observed by yourself, small-pox followed exposure to the contagion; and, *vice versa*, whether in all, or in any considerable proportion, of the cases of small-pox after vaccination observed by yourself, the progress of the vaccine vesicle was *distinctly* ascertained to have been irregular?

"7. Whether, in your opinion, the character of the secondary affection,—in other words, the *degree* to which small-pox is modified when occurring after vaccination,—is derived *solely* from the *degree* of perfection which the vaccine vesicle had put on during its progress?

"8. Whether any thing has occurred to shake your confidence in the power of vaccination to exterminate small-pox altogether, provided it be solely and universally propagated?

"9. Whether the statement made by you (in a letter addressed to a lady near Devizes, and published in many newspapers and journals,) that small-pox is *entirely unknown* in many of the continental kingdoms is derived from authentic *private* sources, or from the published reports of the physicians of those countries, or such other sources of information as are open to the profession generally?

"10. Whether, when you state small-pox to be *wholly* got rid of in those countries, you include the complaints called in this country 'varioloid,' and believed here to originate in, and to be capable of propagating in their turn, the contagion of small-pox?"

My object, Mr. Editor, in putting this series of queries, was to endeavour to draw forth Dr. Jenner's opinions on the

4

important subjects of the causes of vaccine small-pox, revaccination, and the reasonable probability of exterminating small-pox altogether. I added to the queries some observations of my own on these three topics; but, as my intention in the present communication is confined to the laying before your readers the opinions of Dr. Jenner, I have here omitted them. Upon a future occasion, perhaps, I may ask your permission to recur to the subject.

The following was Dr. Jenner's reply:—

"*Berkeley; July* 31, 1821.

"Sir,—I have had the honour of receiving your letter, dated June 22, 1821; for which I beg you to accept my acknowledgments.

"The wishes of great numbers interested in the subject of vaccination with regard to the publication of my further inquiries, will, I hope, be complied with in due time. With a view to extend the general rules necessary for observance in the practice of vaccination, the circular has been published, as you know; and, at the same time, to collect the opinions and observations of others, in addition to my own. After the full operation of this measure, so expedient in the present times, I hope to give the public the results on the most extended basis, as formed by my own researches, and those of others who have particularly attended to the subject. A projected work of this nature would then, as you must fully conceive, be imperfectly anticipated, in its principal bearings, by such replies as must necessarily be given to complete all your queries. I shall have great pleasure, however, in giving a general answer.

1 and 2.—I must observe, that, in forming an accurate judgment of the causes of failure in vaccination, it is absolutely requisite to obtain correct information of the then existing state of the subject, and whether any of the important coincidences which I have made known were influential during its progress. Without such information, (as the operation of that law of the animal economy which produces confusion when the vaccine and herpetic fluids meet through the medium of the constitution, has been witnessed in innumerable instances,) no datum can be formed.

With respect to those dictates which have emanated from the National Vaccine Establishment, I have not, in many points, held myself bound to concur; nor am I to be identified with that body, though by no means at variance. My own opinions are formed on long and almost exclusive physiological attention to the subject; and I have measured their value by the success of my own practice, and that of those who have strictly adhered to the rules arising out of these observations.

I am enabled to reply, that strict attention to the state of the skin, to the uninterrupted progress of the vesicle, and caution as to mechanical interferences, will, as a general law, produce complete protection; though it would be in opposition to my former opinions, if I maintained that variolous susceptibility could in all cases be *entirely* extinguished. (See, in one of the early volumes of the Medico-Chirurgical Transactions, a paper communicated by me, and entitled 'Facts, for the most part unobserved, or not duly noticed, respecting Variolous Contagion.') But, if we admit the occasional influence of small-pox infection upon vaccinated subjects from latent habits of constitution, so we must admit that we are justified by facts in balancing against this the numerous liabilities to recurrence of its own action.

"3d Answer.—I know of none at the present moment.

"4th.—If the vaccine vesicle is in the first instance imperfect, and the impediment is not removed till subsequently, it is open to a second action in different gradations, according to the character which the vaccine vesicle put on in the first instance.* Even though a vaccine vesicle has been sufficient to grant full security, an imperfect pustule may form from very variable dispositions of the skin, and run through its course in a time much limited by the true. The more general consequence, however, is resistance to the restoration of its own action.

"5th.—Failure of well-conducted revaccinations is, according to my experience, decisive, unless the original impediment which had deranged the primary process continues to exist; for it is one of the laws of vesicular affections in such cases occasionally to obstruct any tendency of the vaccine or variolous fluids to re-establish themselves at all, till cleared away. In such cases, no appearance, or the appearance of an irregular vesicle, would be too equivocal to warrant security from the previous vaccination.

"6th.—In considerable numbers, I have seen varioloids occur in all their gradations. To the second part of this query, I may answer in the affirmative; but it is impossible always to procure the requisite information.

"8th.—No. Every thing to increase it in a higher degree, especially since I discovered the grand impediment.

"9th.—Both private and public; from the principal authorities and cities in Europe, as well as the British staff in our possessions in Asia.

"10th.—Varioloids must have occurred every where, where

---

* See my paper, vol. xii. p. 97, *London Medical and Physical Journal*, where perfect failure is shown to have followed a vesicle deranged by herpes.

my rules have not been enough regarded. Continental nations begin now, much to my happiness, to glance at the most common cause.

"The letter to which you allude as lately published at Devizes, was improperly stripped of all its accompaniments by the newspapers which republished it. It came out by the earnest desire of a lady there, and of a gentleman of respectability, who headed it by a note of his own. It was followed by an admirable letter of Dr. Headley's, who is well known as an eminent and very extensive practitioner in that country. Being thus circulated without its explanations, it appeared very different from what I intended; and I wish you to have the goodness to name it to any one who may be struck with its singularity.

"I have the honour to be, Sir,
"Your obedient and very humble servant,
"EDW. JENNER."

"*To Dr. Gregory, London.*"

It is not my intention at present, Mr. Editor, to prosecute this subject farther. Its importance must, I think, be very generally admitted, and may be offered in apology for laying before your readers a correspondence which was not intended for the public eye.

98, *Great Portland-street; August* 5, 1822.

